# Complement regulatory protein Crry deficiency contributes to the antigen specific recall response in experimental autoimmune myasthenia gravis

**DOI:** 10.1186/1476-9255-9-20

**Published:** 2012-05-29

**Authors:** Jindrich Soltys, Xiaobo Wu

**Affiliations:** 1 Department of Neurology & Psychiatry, 1438 South Grand Boulevard, Saint Louis University School of Medicine, Saint Louis, MO, 63104, USA; 2 Department of Medicine, Division of Rheumatology, 660 South Euclid Avenue, Washington University School of Medicine, Saint Louis, MO, 63110, USA

**Keywords:** Acetylcholine receptor (AChR), Experimental autoimmune myasthenia gravis (EAMG), Complement receptor 1-related gene/protein y deficiency (Crry ^−/−^), Adaptive immune response

## Abstract

**Background:**

Myasthenia gravis (MG) and animal model of experimental autoimmune myasthenia gravis (EAMG) is the most common autoimmune disorder of neuromuscular transmission. The disease is caused by the breakdown of the acetylcholine receptor (AChR) which is largely due to complement activation at the neuromuscular junction (NMJ). Limited knowledge exists to the extent that complement receptor 1-related gene/protein y deficiency (Crry ^−/−^) modulates the adaptive immune response and EAMG outcome.

**Methods:**

Mouse EAMG was induced by s.c. administrations of purified acetylcholine receptor (*AChR*) to Crry ^−/−^ and age- matched WT (C57BL/6) mice. Disease severity was assessed by clinical score assessment and muscle grip strength measurements. Serum complement activity was determined by hemolytic assay. ELISA was used to detect the level of AChR specific antibodies. Splenic cells were analyzed for T and B cells subsets distribution, release of cytokines and AChR specific recall responses. Deposition of complement components at the NMJ was assessed by immunofluorescence staining.

**Results:**

In comparison to WT EAMG, Crry ^−/−^ EAMG mice showed signs of augmented muscle weakness but differences, except for one time point, were not statistically significant. Serum complement activity was reduced in Crry ^−/−^ EAMG mice and no substantial changes in deposition of C3, C3b/iC3b and C5b-9 (MAC) at the NMJ between WT EAMG and Crry ^−/−^ EAMG mice were detected. Lack of Crry affected adaptive immune response. Crry ^−/−^ EAMG mice showed increases in the number of AChR specific splenic T-cells secreting IFN-γ and IL-4. Production of complement fixing antibodies (IgG_2b,_ IgG_2c_) was also augmented. More Th1, Th2 and Th17 cytokines were released into the bloodstream of Crry ^−/−^ EAMG mice.

**Conclusions:**

Data suggest that Crry deficiency modulates the adaptive immune response in EAMG, but its effect on disease outcome is limited. This was due to the generally lower serum complement level caused by increased C3 turnover. Modulation of complement activity with soluble or membrane bound regulators of complement activity represents a potentially effective approach to modify autoimmune processes in MG and EAMG.

## Background

Myasthenia gravis (MG) is an autoimmune disorder in which autoantibodies against nicotinic acetylcholine receptors (AChR) trigger the destruction of the neuromuscular junction (NMJ). Three distinct mechanisms are proposed to explain neuromuscular transmission failure [1]. The AChR breakdown can be caused by a direct block of receptor function which is primarily due to antibodies that recognize the binding site for the cholinergic ligand. The other pathological mechanisms are pointed to enhanced endocytosis and degradation of the AChR triggered by antibody crosslinking, and complement mediated lysis of the NMJ.

The leading role for complement involvement in MG pathogenesis is supported by multiple studies [2-4]. MG patients show increased deposits of C3 and membrane attack complex (C5b-9; MAC) at the NMJ [5-7] and amplified complement consumption *in vivo* has also been observed [8]. The simplified NMJ structure in MG is a more likely consequence of complement mediated injury [9].

The complement system plays an important role in innate immunity and mediates crosstalk between innate and adaptive immunity [10]. There is a delicate balance *in vivo* between complement activation and its inhibition. If this equilibrium is altered, the complement system causes tissue injury and contributes to the pathogenesis of various diseases [11], including neurodegenerative disorders and other neuropathies [12]. Therefore, complement activation is strictly controlled by the regulators of complement activity (RCA). Both membrane bound and soluble RCA have the capacity to prevent the exaggerated complement activation [13].

Limited knowledge is available regarding how specific RCA affect the outcome of experimental autoimmune myasthenia gravis (EAMG). In this study, we examined the effect of complement receptor 1-related gene/protein y deficiency (Crry ^−/−^) on EAMG pathogenesis. Rodent specific Crry has similar regulatory functions as human decay accelerating factor (CD55/DAF) and membrane cofactor protein (CD46/MCP) [14,15]. Crry is the only ubiquitously expressed transmembrane protein with cofactor activity which is essential to control activation of C3 complement component and protect self-tissues from complement mediated lysis [16]. Crry ^−/−^ mice experience uncontrollable alternative pathway (AP) turnover in their plasma leading to an approximately 60% reduction of serum C3 and factor B (fB). However, the magnitude of AP mediated-complement consumption in Crry ^−/−^ is less severe than those in fH^−/−^ mice in which over 90% of serum C3 was consumed in mice missing this fluid phase complement regulator [17]. In contrast to the spontaneous development of dense deposit glomerulonephritis in fH^−/−^ mice, no renal pathology is documented in Crry ^−/−^ mice [14,18]. In addition, Crry also has been shown to protect cells from complement attack and is involved in T cell co-stimulation [19].

Based on previously described Crry regulatory properties, we hypothesized that lack of Crry in mice with EAMG would lead to a more severe disease outcome. The rationale was to examine the importance of the Crry deficiency on EAMG pathology by comparing the clinical and immunological aspects of the disease in RCA sufficient WT control (C57BL/6) and RCA deficient Crry mice (Crry ^−/−^). Our data show that Crry deficiency had a direct impact on humoral and adaptive immune responses. However, lack of Crry did not augment significantly disease severity *in vivo*.

## Materials and methods

### Mice

Crry ^+/−^ mice were generated by a standard gene targeting approach and were backcrossed into C57BL/6 background for over eight generations. Initial observations showed that survival of Crry null embryos is compromised due to the uncontrollable complement activation and concomitant placenta inflammation [20]. In order to obtain viable Crry ^−/−^ mice (also called Crry single knockout mice, Crry SKO), we utilized strategic breeding using female breeding partners with impaired complement (AP) capacity [14]. Crry ^−/−^ genotyping was performed by PCR with primers of mCrry 16 TTGAGTTCAATGCACTGAGGAGG, *Eco*RI 16 F CGCAGAATTCAATCTCTTTTCT TTGCC and S46Neo GCTACCCGTGATATTGCTGAAGAG. Wild-type (WT, C57BL/6) mice were purchased from the Jackson Laboratory (Bar Harbor, ME). Mice were housed and maintained in a pathogen-free condition at the Saint Louis University Department of Comparative Medicine. All experiments were performed according to the protocols approved by SLU IACUC.

### Induction and clinical evaluation of experimental autoimmune myasthenia gravis (EAMG)

The acetylcholine receptor was purified from the electric organs of *Torpedo californica* (*tAChR*) by affinity chromatography [21]. Eight to ten weeks old WT and Crry ^−/−^ male mice were used for experiments. EAMG was induced by four subcutaneous injections of 20 μg *tAChR* emulsified in complete Freund’s adjuvant (CFA) (Difco, Voigt Global Distributions, KS) in a total volume of 200 μl. Mice were immunized along the back subcutaneously, at the base of the tail and boosted twice with 20 μg of *tAChR* in incomplete Freund’s adjuvant 4 and 8 weeks after primary immunization. Control mock immunized mice received an equal volume of PBS in CFA or IFA.

To validate disease induction in WT and Crry ^−/−^ mice were bled ten days after primary and secondary immunization. Sera were collected and screened by ELISA for the production of AChR specific antibodies. EAMG outcome was assessed on a weekly basis. All mice were clinically scored [22], weighed and examined for muscle weakness. Measurements were performed with a grip strength meter (Columbus Instruments, Columbus, OH) and DFE digital force gauge (Ametek, Largo, FL) was used to detect the peak force when animals grasp a grid pull bar. Prior to the measurement, each mouse was exercised with 10–20 paw grips and then the final 5 grips were recorded and analyzed.

### Complement activity

Complement activity in WT EAMG, Crry ^−/−^ EAMG and mock immunized WT Ctrl, Crry ^−/−^ Ctrl mice were analyzed one week after the primary or secondary immunization (Day 8, 35) and at the end of experiment (Day 63). Serum diluted 1:10, 1:20, 1:40 and 1:80 in Veronal buffer was analyzed by a CH_50_ hemolytic assay according to the manufacturer’s protocol (Sigma, St. Louis, MO). Briefly, 100 μl of 2 × 10^8^ sensitized sheep erythrocytes were mixed with pre-diluted serum and then incubated for 60 minutes at 37°C. At the end of the incubation time, un-lysed cells were removed by centrifugation (Sorvall Legend RT^+^ benchtop centrifuge: 1500 RPM for 5 minutes) and the intensity of complement mediated hemolysis was measured at 412 nm on Tecan Infinity M200 reader (Tecan Group Ltd., Durham NC).

### ELISA for AChR specific IgG subclasses

Conventional ELISA was used for the detection of anti-AChR specific complement fixing antibodies. Serum levels of AChR antibodies were examined at Days 10, 35 and 63 post primary immunization (p.i.). A 96 well Nunc plates (Fisher Scientific, Pittsburgh, PA) were coated overnight at 4°C with 10 μg/ml of purified AChR (100 μl/well). After three washes with PBS-Tween, the plates were blocked for 2 hrs at room temperature (RT) with 200 μl PBS Tween 20 (Sigma, Saint Louis, MO). Mouse serum samples in triplicates at dilution of 1:500 were added (100 μl/well) and incubated at RT for 90 minutes. After washes with PBS-Tween, the plates were incubated for another 90 min with HRP conjugated goat anti-mouse Abs (IgG, IgG_1_, IgG_2b_, IgG_2c_; 1:2000; Alpha Diagnostics, San Antonio, TX). The color reaction was developed with SureBlue TMB substrate and stopped with TMB stop solution (KPL Inc., Gaithersburg, MA). Stopped reactions were read on a Tecan Infinity M200 reader (Tecan Group Ltd., Durham NC). Absorbances were measured at 450 nm and the results were expressed in O.D. values.

### Immunofluorescence detection of C3, C3b/iC3b and C5b-9 (MAC) complement components

Mouse diaphragms were embedded in OCT Compound Tissue-Tek (Fisher Scientific, Pittsburgh, PA) and were frozen in liquid N_2_-cooled 2-methybutane. Tissue samples were stored at −80°C until usage. For IHC analysis of C3, C3 fragments (C3b/iC3b/C3c) and C5b-9 deposition at the NMJ, 10 μm cryosections of mouse diaphragms were mounted on Superfrost^Plus^ slides. Slides were allowed to air dry and tissues were fixed in cold acetone for 5 minutes. After three washes with PBS, sections were blocked with 3% BSA in PBS for at least 1 hour. Tissues were further stained with FITC conjugated anti-mouse C3 antibody (MP Biomedicals, Solon, OH). For recognition of C3b/iC3b/C3c rat anti-mouse monoclonal antibody (clone 3/26; Hycult Biotech, Plymouth Meeting, PA) and rabbit anti-mouse C5b-9 (EMD Biosciences, San Diego, CA) polyclonal antibodies were used. Although antibody 3/26 recognizes C3b/iC3b/C3c, soluble C3c is not present on the cell surfaces [23]. All these primary antibodies were diluted at 1:200 and 1:300, respectively. For C3b/iC3b and C5b-9 (MAC) staining, sections were labeled with Alexa^488^ conjugated goat anti-rat and goat anti-rabbit secondary antibodies (1:500; Invitrogen, Carlsbad, CA), respectively. Finally, Alexa Fluor^596^ labeled bungarotoxin (1:1000; BTX, Invitrogen, Carlsbad, CA) was used to visualize the NMJ. After washes, sections were viewed by an Olympus fluorescence microscope (Olympus Inc, USA). Captured microphotographs were analyzed with Image Pro software (Media Cybernetics, Silver Springs, MD). Results were expressed as percentage of C3 fragments and C5b-9 deposits present at the BTX labeled NMJs.

### ELISPOT

Plates coated with primary capture antibodies specific for IFN-γ and IL- 4 from BDTM Elispot Kits (1:200; BD Biosciences, San Jose, CA) were used for detection of cytokine secreting cells. Single cell suspensions (5 × 10^5^/well) of splenocytes in 100 μl of complete RPMI-1640 medium were added in triplicate and incubated for 24 hours, with or without recall antigen of *tAChR* (at the concentrations of 10; 1.0; 0.1 and 0.01 μg/well). After washes with PBS-Tween buffer, cells were stained overnight with secondary biotin labeled anti-IFN-γ and IL-4 antibodies. After three washes with PBS-Tween buffer, streptavidin-HRP was added and plates were incubated for 60 minutes. ELISPOT plates were developed with BDTM ELISPOT AEC substrate sets (BD Biosciences, San Jose, CA). Spots were counted on Immunospot Image Analyzer using Beta 4.0 version software (ELISPOT Image Analyzer, Cellular Technology Ltd., Cleveland, OH).

### FACS analysis

Distribution of T and B cells subsets was analyzed by Becton Dickinson FACSCalibur flow cytometer or FACSArray Bioanalyzer according to the manufacturer’s protocol (BD Biosciences, San Jose, CA). Splenic cell suspensions were harvested at Day 63 post primary immunization and stained with antibodies specific for T (CD3ε PE-Cy7, CD4 PE, CD8 APC) and B (CD45R/B220 PE-Cy7, CD23 PE, sIgM APC) cell subsets markers (BD Biosciences, San Jose, CA). Data were analyzed with WinlistTM software (Verity Inc., Sunnyvale, CA).

### Cytometric beads array (CBA)

The BDTM CBA Mouse Th1/Th2/Th17 Cytokine Kit (BD Biosciences, San Jose) was used to measure IL-2; IL-4, IL-6, IFN-γ, TNF and IL-17 protein levels. The procedure was carried out according to the manufacturer's protocol (CBATM, BD Biosciences, San Jose, CA). Serum samples from individual mice were collected at Day 63 p.i. Total 25 μl of serum was mixed with 25 μl of assay diluent. Then, 50 μl of cytokines capture beads and 50 μl of PE-labeled detection antibody were added to each diluted serum and incubated for 2 hrs at RT. Cytokine standard solutions from the BDTM CBA Kits were diluted from concentrations of 0 to 5000 pg/ml. After incubation in the dark at RT for 2 hours, all cytokine standards and samples were washed twice with buffer. Finally, bead pellets with captured cytokines were resuspended in 300 μl of wash buffer and read on BD FACSArray Analyzer (BD Biosciences, San Jose, CA). Acquired data were further analyzed with FCAP ArrayTM software (Soft Flow Inc. Minneapolis, MN).

### Statistical analysis

To compare all groups of data, experimental groups were evaluated by a Two-way ANOVA followed by the Bonferroni post hoc test. Column analyses were performed by Mann - Whitney test. Data are presented as the mean ± SEM where P values < 0.05 are considered statistically significant. Data analysis was performed using GraphPad Prism version 5.04 for Windows (GraphPad Software, San Diego, California, USA).

## Results

### Development and progression of disease in WT EAMG and Crry ^−/−^ EAMG mice

**Figure 1:**
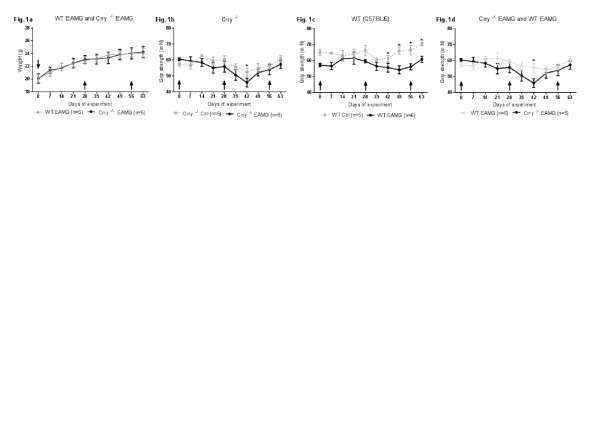
**a, b, c, d. Deficiency of Crry**^**−/−**^**did not alter extensively the clinical outcome of EAMG.** Mice were immunized with 20 μg of *tAChR* or PBS at the times indicated with arrows (↑). Weight loss (Figure 1**a**) and muscle grip strength (Figure 1**b**, **c**, **d**) measurements were used to assess the clinical outcome of EAMG. See *Material and Methods* for experimental details. Data shown are one of three independent experiments. Error bars indicate ± SEM values; n = 5–9 mice per group. Asterisks (*) on top of the time points indicate statistically significant differences between WT EAMG and Crry ^−/−^ EAMG mice (p < 0.05).

To permit satisfactory statistical analysis, disease outcome in WT EAMG and Crry ^−/−^ mice was determined by three independent experiments with 5–9 mice in each experimental group. Mouse weight and grip strength in WT EAMG, Crry ^−/−^ EAMG (*tAChR*) and mock (PBS) immunized controls of WT, Crry ^−/−^ mice were assessed for the period of 9 weeks after the primary immunization. We did not detect significant changes in weight loss between WT EAMG and Crry ^−/−^ EAMG mice (Figure 1a). Muscle grip strength varied between WT and Crry ^−/−^ mice. In general, Crry ^−/−^ EAMG mice (Figure 1b) appeared to be weaker than their WT EAMG counterparts (Figure 1c). Crry ^−/−^ mice showed signs of augmented muscle weakness in an EAMG but the differences, except for one time point (Day 42 p.i.), were not statistically significant (Figure 1d). In addition, Crry ^−/−^ EAMG mice exhibited no acceleration in disease progression. Furhermore, clinical score analysis correlated with our muscle grip strength data in which no differences between RCA deficient (Crry ^−/−^ EAMG) and RCA sufficient (WT EAMG) mice (Table 1) were observed.

**Table 1:** **WT EAMG and Crry**^**−/−**^**EAMG mice show similar disease outcome**

**Mouse strain**	**Day 0**	**Day 14**	**Day 35**	**Day 63**
WT EAMG	0	0.400 ± 0.22	1.000 ± 0.35	1.300 ± 0.57
Crry ^−/−^ EAMG	0	0.375 ± 0.25	0.875 ± 0.25	1.250 ± 0.50
WT Ctrl	0	0	0	0
Crry ^−/−^ Ctrl	0	0	0	0

EAMG clinical scores were determined at Day 0 (prior disease induction), Days 14, 35 and 63 post primary immunization. The data for each mouse strain represent the average (± SEM) in one of three representative experiments (n = 5–9 mice per group). Despite greater general weakness in Crry ^−/−^ EAMG, no significant changes in disease clinical scores were detected.

### Serum complement activity

**Figure 2:**
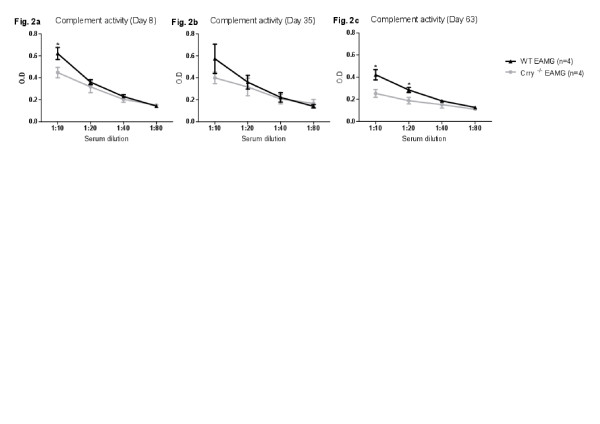
**a, b, c. Crry**^**−/−**^**mice have decreased complement activity during actively induced EAMG.** Complement activity was measured at Day 8, 35 and 63 (Figure 2**a**, **b**, **c**) by complement hemolytic assay. See *Material and Methods* for experimental details. Data is representative of three independent experiments. All error bars are ± SEM. Asterisks (*) on top of the time points indicate statistically significant differences between WT EAMG and Crry ^−/−^ EAMG mice (p < 0.05).

Crry protein has been shown to regulate classical and alternative complement pathways [24] and Crry ^−/−^ mice have reduced C3 and fB concentrations due to complement consumption through uncontrolled complement alternative pathway (AP) turnover [14]. A standard hemolytic assay was employed to measure complement activation of WT EAMG and Crry ^−/−^ EAMG mice. Examining WT EAMG and Crry ^−/−^ EAMG serum at dilutions 1:10; 1:20; 1:40; 1:80, collected at different days after disease induction (Day 8, 35 and 63 p.i.), we observed that total complement activity in Crry ^−/−^ EAMG mice at dilution 1:10 was about 20-40% lower when compared to the complement regulator sufficient WT EAMG mice. Complement levels in Crry ^−/−^ EAMG mice remained low at Days 8 and 35 (Figure 2a, b). At day 63 p.i. WT EAMG and Crry ^−/−^ EAMG mice showed reduced complement titers (Figure 2c). Disease induction in Crry ^−/−^ EAMG mice did not augment complement activity in blood serum. This was possibly due to a complex issue where in Crry ^−/−^ mice an uncontrolled complement turnover and a major membrane complement regulator deficiency exists at the same time.

### Levels of AChR specific IgG antibodies

**Figure 3:**
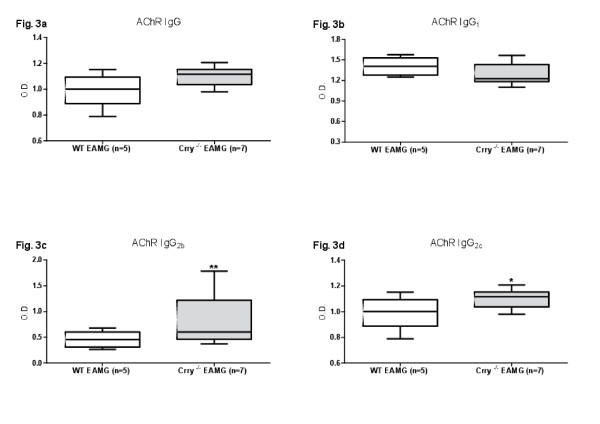
**a, b, c, d. The level of anti-AChR specific complement fixing antibodies IgG**_**2b**_**and IgG**_**2c**_**is augmented in Crry**^**−/−**^**EAMG mice.** The amount of antibodies produced was determined by ELISA and the level of anti-AChR antibodies production is expressed in O.D. Blood serum from WT EAMG and Crry ^−/−^ EAMG mice was collected at Day 63 p.i. Data shown represent one of three independent experiments. Error bars indicate ± SEM values; Asterisks on the top of the bars (*, **) represent statistically significant difference at p < 0.05 and p < 0.01.

Next, we examined the effect of Crry deficiency on the production of anti-AChR antibodies where the levels of specific antibody subclasses (IgG, IgG_1_, IgG_2b_, IgG_2c_) were measured by ELISA at Day 10, 35 and 63 post primary immunization (p.i.). As shown on Figure 3a (Day 63 p.i.), the total amount of anti-AChR IgG antibody was elevated in Crry ^−/−^ EAMG. While the IgG_1_ levels were comparable between the WT and Crry ^−/−^ groups (Figure 3b), the production of complement fixing antibodies IgG_2b_ (Figure 3c) and IgG_2c_ (Figure 3d) in Crry ^−/−^ EAMG mice was significantly increased. Similar patterns in the AChR specific antibodies profile, but to a lesser magnitude, was observed on Day 10 and seven days after the secondary immunization (AChR + IFA; Day 35 p.i.). Mock immunized mice (PBS + CFA or PBS + IFA) did not generate any measurable levels of anti-AChR antibodies.

### Immunofluorescence staining for the deposition of C3, C3 fragments (C3b/iC3b) and C5b-9 (MAC) at the NMJs

**Figure 4:**
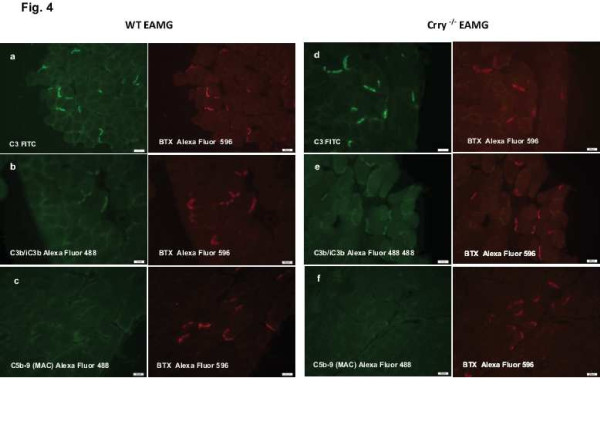
**a, b, c, d, e, f. Decreased complement activity in blood serum of Crry**^**−/−**^**EAMG mice did not alter the amount of complement deposition at the NMJs.** Diaphragms from WT EAMG (Figure 4**a**, **b**, **c**) and Crry ^−/−^ EAMG (Figure 4**d**, **e**, **f**) mice were stained for the presence of C3, C3b/iC3b and C5b-9 (green fluorescence) complement components. The NMJs were visualized with bungarotoxin (BTX; red fluorescence). Not immunized WT Ctrl and Crry ^−/−^ Ctrl mice did not show complement deposition at the NMJ (data not shown). Diaphragms of a minimum of 5 mice from each experimental group were analyzed. Scale bars for magnification (20 μm) are shown on each microphotograph. See Material and Methods for experimental details.

**Figure 5:**
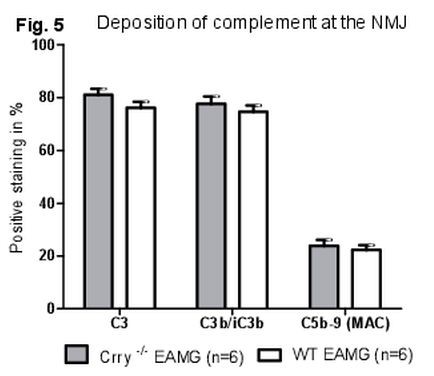
**Deposition of C3, C3b/iC3b and C5b-9 (MAC) is similar at the NMJs of WT EAMG and Crry**^**−/−**^**EAMG mice.** Diaphragms from WT EAMG and Crry ^−/−^EAMG mice were stained for C3, C3b/iC3b and C5b-9 (MAC) and BTX staining was used to visualize the NMJs. The results are expressed as percentage of NMJ (%) with detected complement deposits. Minimum three sections with 10–15 NMJs from each diaphragm were quantified (n = 5 mouse per experimental group).

As a result of augmented production of complement fixing antibodies (IgG_2b_, IgG_2c_) in Crry ^−/−^ EAMG mice, a significant deposition of IgG and subsequent complement activation at the NMJs were expected. Complement deposition at the NMJs was assessed by staining for C3, C3 fragments (C3b/iC3b) and C5b-9 (MAC). Despite the overall lower complement activity in blood serum of Crry ^−/−^ mice, diaphragms from WT EAMG and Crry ^−/−^ EAMG showed similar deposition of C3 (Figure 4a, b) and C3 fragments (C3b/iC3b; Figure 4d, e). Interestingly, strong presence in complement components at the NMJs of WT EAMG and Crry ^−/−^ EAMG mice did not result in effective MAC formation (C5b-9; Figure 4c, f). Quantitatively, we did not find any significant differences in deposition of C3, C3b/iC3b fragments and C5b-9 (MAC) at the NMJ of WT EAMG and Crry ^−/−^ EAMG mice (Figure 5). Mock (PBS) immunized WT and Crry ^−/−^ mice did not exhibit any IgG or complement components deposition at the NMJ (data not shown).

### Specific recall responses to the AChR antigen

**Figure 6:**
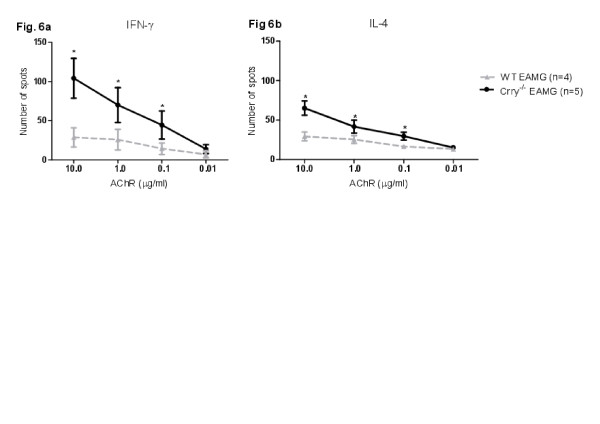
**a, b. Crry**^**−/−**^**EAMG mice show augmented IFN-γ and IL-4 recall responses to*tAChR ex vivo*.** Splenic cell suspensions (10^5^ cells/well) isolated from WT EAMG and Crry ^−/−^ EAMG mice were re-stimulated in triplicate with *tAChR* at concentrations of 10, 1.0, 0.1, and 0.01 μg/ml. The frequency of IFN-γ and IL-4 secreting cells was determined by ELISPOT assay and additional controls for negative or positive spot production were established. Con A (1 μg/ml) treated cells (positive control) generated comparable number of spots in all experimental groups (243 ± 57 WT and 211 ± 36 Crry ^−/−^), whereas PBS immunized (negative control) mice did not have any spots. Results shown are representative of two independent experiments. Asterisks (*) represent statistically significant differences at p < 0.05.

Furthermore, we investigated the effect of Crry deficiency on the development of the adaptive immune response in actively induced EAMG. The recall response of splenic T cells to the AChR antigen was determined by ELISPOT assay on Day 63 post primary immunization. At this time point, the frequency of *tAChR* responding cells isolated from WT EAMG and Crry ^−/−^ EAMG was considerably different. Crry ^−/−^ EAMG mice have more IFN-γ (Figure 6a) and IL-4 (Figure 6b) positive cells than WT EAMG mice. The numbers of spots were increased in a dose dependent fashion when recall antigens were provided at a dose of 10.0, 1.0, 0.1 μg/ml of *tAChR*. Re-stimulation of control (PBS) immunized Crry ^−/−^ and WT cells with *tAChR* showed no spot formation. Stimulation with Con A (1 μg/ml) resulted in development of a comparable number of spots in both complement regulator deficient (Crry ^−/−^) and complement regulator sufficient WT mice (data not shown).

### T and B cells subsets distribution in WT EAMG and Crry ^−/−^ EAMG

**Figure 7:**
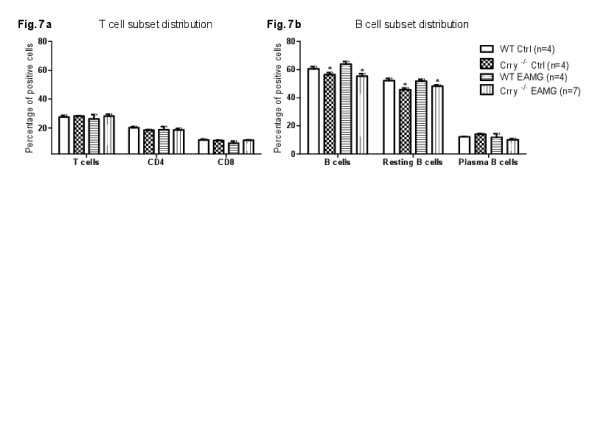
**a, b Induction of EAMG in Crry**^**−/−**^**and WT mice did not affect the distribution of T and B cell subsets.** Splenic cell suspensions from *tAChR* and mock immunized mice were analyzed by Flow cytometry at Day 63. Results are expressed as percentage of positive cells for each marker (CD3ε PE-Cy7, CD4 PE, CD8 APC, CD45R/B220 PE-Cy7, CD23 PE, sIgM APC; BD Biosciences). Results shown are representative of two independent analyses (n = 4–7 mice per group).

We have also examined if established EAMG affects T and B cell subset distribution in Crry ^−/−^ mice. T and B cell subset analysis was performed in WT EAMG and Crry ^−/−^ EAMG mice by flow cytometry at Day 63 post immunization. As a control, we also included WT and Crry ^−/−^ mice without disease induction. WT EAMG and Crry ^−/−^ EAMG mice had comparable T cell subset distribution (Figure 7a). Repeated immunization with *tAChR* did not have direct effect on T and B cell subset distribution (Day 63). However, the percentage of B cells in the spleen of Crry ^−/−^ mice was reduced (Figure 7b). Decline in B cell population was not related to the EAMG.

### Th1, Th2 and Th17 cytokine production in EAMG

**Figure 8:**
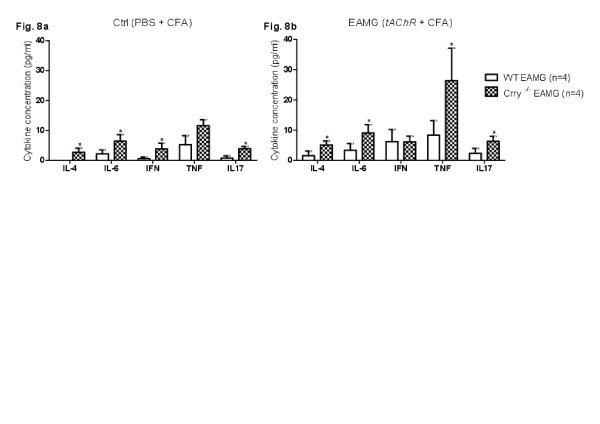
**a, b. Levels of Th1, Th2 and Th17 cytokines are increased in plasma of Crry**^**−/−**^**EAMG. ***Ex vivo* cytokine production from blood plasma collected at Day 63 p.i. from mock immunized WT and Crry ^−/−^ mice and *tAChR* immunized mice (WT EAMG and Crry ^−/−^ EAMG) was analyzed with cytometric bead arrays (CBA, BD Biosciences). Individual cytokine concentrations are shown in pg/ml. Asterisks (*) indicate values p < 0.05 between complement regulator sufficient WT and complement regulator deficient Crry ^−/−^ mice (Figure 6a - Ctrl, Figure 6b - EAMG). Results shown are representative of three independent analyses (n = 4–6 mice per experimental group).

We investigated whether there were any differences in production of Th1, Th2 and Th17 cytokines followed by *tAChR* immunization. While only pro-inflammatory cytokines (IL-6 and TNF) were detected in WT Ctrl mice (Figure 8a), blood serum of Crry ^−/−^ Ctrl mice showed small increases in levels of IL-2, IL-4, IL-6, IFN-γ and IL-17. In comparison to the WT Ctrl, Crry ^−/−^ Ctrl and WT EAMG the highest serum cytokines concentrations were detected in Crry ^−/−^ EAMG mice (Figure 8b). Crry deficiency in EAMG was associated with increased serum levels of the pro-inflammatory TNF as well as IL-2, IL-4, IL-6 and IL-17. In average Crry ^−/−^ EAMG mice showed about two to three folds increase in serum cytokines concentration (Table 2). We assume that increase in IL-6 and TNF cytokine levels in WT Ctrl mice was caused by repeated immunizations with complete and incomplete Freund’s adjuvant (PBS + CFA, PBS + IFA).

**Table 2:** **Cytokine induction ratio is increased in Crry**^**−/−**^**EAMG mice**

**Cytokine (fold increase)**	**TNF**	**IL-4**	**IL-6**	**IL-17**
Crry ^−/−^ EAMG / WT EAMG	3.17	3.27	2.71	2.74

Crry deficiency in EAMG was associated with increased serum levels of the pro-inflammatory TNF as well as IL-4, IL-6 and IL-17. Cytokine induction ratio (fold increase) in Crry ^−/−^ EAMG group was calculated as follows. An average serum cytokine concentration in Crry ^−/−^ EAMG was divided by serum cytokine concentration in WT EAMG mice (Day 63 post primary immunization).

## Discussion

Our study shows that lack of Crry in EAMG is associated with altered humoral and adaptive immune responses. Crry deficiency enhanced the production of complement fixing antibodies (IgG_2b_, IgG_2c_) and augmented specific recall responses *in vitro*. Production of Th1, Th2 and Th17 cytokines was also increased. In comparison to WT EAMG, Crry ^−/−^ EAMG mice showed symptoms of induced muscle weakness but this difference, except one time point, did not reach a statistical significance. This result was probably due to a unique situation where simultaneously Crry deficiency and reduced amount serum C3 and fB levels occur in Crry ^−/−^ mice.

Analysis of splenocytes from Crry ^−/−^ EAMG mice demonstrated that there were significant changes in the frequency of IFN−γ and IL-4 secreting cells after the re-stimulation with *tAChR*. It is well established that IFN-γ and IL-4 play a critical role in the pathogenesis of EAMG. IFN-γ knockout mice showed dramatic reduction in mouse AChR-specific IgG_1_ and IgG_2a_ antibodies and were resistant to EAMG [25]. A recent study on rat myocytes suggests that the IL-4 receptor provides a link between the immune system and muscle in EAMG [26]. Overproduction of IL-6 also plays an important role in age related pathogenic mechanisms mainly in early-onset of MG [27]. This corroborates with the evidence of steroids preventing MG crisis through their effect on down-regulating IL-6 [28]. In summary, pro-inflammatory cytokines affect the *AChR* expression and contribute to the initiation of the autoimmune response [29]. We found that levels of IL-2, IL-4, IL-6, TNF, and IL-17 cytokines were elevated in serum of Crry ^−/−^ EAMG mice. Similarly to Mu et al. we showed that the cytokine balance is rearranged during disease development and IL-17 is involved in EAMG [30].

However, increased number of IFN-γ and IL-4 cells in Crry ^−/−^ EAMG had a restricted impact on the clinical outcome of EAMG in our current study. In comparison to the previous studies that EAMG model was performed on a C3 sufficient background, we assume that this was due to decreased complement activity in Crry ^−/−^ mice affecting both innate and adaptive immune responses. Despite lowered complement activity in bloodstream of Crry ^−/−^ mice, we observed that there was considerable deposition of C3 and cleaved C3 fragments (C3b/iC3b) at the NMJ of Crry ^−/−^ EAMG mice. If Crry ^−/−^ mice have a normal level of complement, the uncontrolled complement activation would result in a more severe form of tissue injury.

Our current observation and the studies by Heeger et al [31] on decay accelerating factor (DAF) deficient mice support the idea that regulators of complement activity impact adaptive immune response [32]. Their significant finding is that the enhanced production of IL-2 and IFN-**γ** by DAF ^−/−^ T cells after re-stimulation is largely complement dependent. Pavlov and colleagues reported similar findings that splenic cells from DAF deficient mice proliferated more vigorously following *in vitro* stimulation with allogeneic cells [33]. The essential role of both complement regulators, Crry and DAF in preventing of tissue injury was validated *in vivo* on antibody induced autoimmune glomerulonephritis [34].

The important role of RCA complement regulatory proteins in EAMG pathogenesis is probably through their functions on altering adaptive immune responses [35,36]. Results from our study show that Crry deficiency modulates antigen specific response in EAMG. However, the detected changes in humoral and adaptive immune response did not eventually lead to the development of an augmented severe disease phenotype. This is in contrast with studies when mice overexpressing Crry or a soluble form of Crry (Crry-Ig) were used for inhibition of complement activity and disease prevention [37,38].

Currently, it is still a challenge for us to fully understand to what extent and how deficiency in specific RCA affects humoral and adaptive immune response in MG and EAMG. Ours and the others data suggest that there is systemic and indirect effect of complement on T cell immunity [39]. Additional evidence indicates that different expression of RCA in muscle could affect the outcome of EAMG. In passively induced EAMG, the expression of Crry and DAF is increased at diaphragm junctions, whereas more DAF and less of Crry is present at the extraocular muscle junctions. This distinctive pattern in Crry and DAF distribution may contribute to the higher susceptibility of eye muscle to MG [40].

## Conclusions

Our observations point to two important biological consequences linked with Crry deficiency and EAMG pathogenesis. Crry ^−/−^ mice have impairment in membrane -related inhibition of complement activation and show failure in controlling complement homeostasis. However, Crry participates in crosstalk between the innate and adaptive immune response. We propose that modulation of complement activity with different soluble or membrane bound regulators of complement activity represents a potentially effective way to modify autoimmune processes in MG and EAMG.

## Abbreviations

EAMG: Experimental autoimmune myasthenia gravis; MG: Myasthenia gravis; RCA: Regulators of complement activity; Crry ^−/−^: Complement receptor 1-related gene/protein y deficient mice.

## Competing interests

The authors declare that they have no competing interests.

## Authors’ contributions

JS and XW contributed equally to the manuscript. Both authors read and approved the final manuscript.
